# Enhancing Data Science and Genomics Capacity of a Historically Black Medical College Through Interdisciplinary Training and Research Collaborations

**DOI:** 10.26502/jbb.2642-91280166

**Published:** 2024-10-18

**Authors:** Qingguo Wang, Vibhuti Gupta, Aize Cao, Pandu Gangula, Hua Xie, Rajbir Singh, Todd Gary, Samuel E Adunyah, Aramandla Ramesh, Anil Shanker

**Affiliations:** 1 Department of Biochemistry, Cancer Biology, Neuroscience and Pharmacology, School of Medicine, Meharry Medical College, Nashville, TN, USA; 2 Department of Computer Science and Data Science, School of Applied Computational Sciences, Meharry Medical College, Nashville, TN, USA; 3 Department of Biomedical Data Science, School of Applied Computational Sciences, Meharry Medical College, Nashville, TN, USA; 4 Department of ODS & Research, School of Dentistry, Meharry Medical College, Nashville, TN, USA; 5 The Office for Research and Innovation, Meharry Medical College, Nashville, TN, USA

**Keywords:** Data science in medicine, Computational genomics, HBCU, Workforce diversity, Health disparities

## Abstract

As data grows exponentially across diverse fields, effectively leveraging big data has become increasingly crucial. In data science and computational genomics, however, minority groups, including African Americans, are significantly underrepresented, coupled with the lack of resources and infrastructure in minority-serving institutions. This paper summarizes the second phase of our funded project that aims to enhance the data science capacity of Meharry Medical College (MMC), a Historically Black College/University (HBCU), by providing training and fostering collaborations between data scientists and researchers in basic science and biomedical fields. Using diverse training approaches and formats, we introduced data science and computational genomics to hundreds of MMC researchers and students in the past 2 years. The training modules designed for dental curriculums introduced artificial intelligence and machine learning to ~250 dental students, 80% of which are African Americans (AA). We have also fostered partnerships between data scientists and other MMC researchers for joint publications and grant applications in various areas that impact the health of AA population. The multiple grants awarded recently to MMC clearly indicate an enhanced data science and genomics capacity of MMC and the impact of our work on the local community.

## Introduction

With an unprecedented amount of data generated in business, sciences, medicine, social media, and healthcare systems, data analyst skills are becoming increasingly crucial for leveraging big data to gain a competitive edge, accelerate scientific discovery, and advance public health [1]. Accordingly, data science has undergone the fastest growth in the past decade. Its widespread applications have transformed industries and the economy, contributed significantly to scientific discoveries [1–4], and raising a competent workforce [5–8]. In particular, the increasing use of data infrastructure, health informatics, and new analytic methods (in artificial intelligence [9–12] and machine learning [13–15]) has led to significant benefits to health and health care: cost-effective drug discovery, personalized medicine, improved patient outcomes and delivery of patient care, etc.

In computing and information disciplines, however, African Americans (AA), along with other minority groups, are underrepresented [16,17], coupled with the lack of resources and infrastructure in minority-serving institutions (MSIs). This paucity is evident in academic admission, employment sectors, and industry [16,17]. To enhance the data science capacity of MSIs and develop a diverse data science workforce, the National Institute on Minority Health and Health Disparities (NIMHD) released a Notice of Special Interest (NOSI) entitled “Administrative Supplements to Enhance Data Science Capacity at NIMHD-Funded Research Centers in Minority Institutions (RCMI)” in May 2021. We as faculty at Meharry Medical College (MMC), in partnership with the RCMI program at MMC, applied for and received the award (Award Number U54MD007586–35S5) in July 2021 and, thus, started this project in September 2021 [18,20,24]. It may be worth noting that MMC is an Historically Black College/University (HBCU). Founded in 1876, it is the first medical school for AAs in the South of the US. Dedicated to educating healthcare professionals and biomedical scientists, it has trained approximately 40% of AA dentists and 8% of AA physicians in the US, the majority of whom work in underserved rural or urban communities. The RCMI Program in Health Disparities Research at MMC is a long-term NIMHD-funded endeavor that enables high-quality basic, behavioral, and clinical research to eliminate health disparities [21–23]. The corresponding U54 award (Award Number U54MD007586) supports health disparities research in a variety of diseases, such as cardiovascular diseases, cancer, diabetes, HIV/AIDS, and neurological diseases that disproportionally affect the non-Hispanic Black population in the US [21–23].

The year one of this NIMHD-funded project (for the period of 09/01/2021 to 09/22/2022) provided data science training through the data science courses offered by MMC’s School of Applied Computational Sciences (SACS) [20,24], whose curriculum and class schedule (offered after 5:30PM), however, do not accommodate most medical/dental/graduate students and faculty, hindering their participation. In year two (September 2022 ~ May 2023, with an authorized carryover to May 2024), we adjusted our strategy by focusing on designing and integrating data science modules for other MMC disciplines. In this period, with MMC’s launch of the Together for CHANGE (T4C) Initiative in late 2023, where CHANGE stands for ‘Changing Health Care for People of African-Ancestry through an International Genomics and Equity Initiative’, we intentionally incorporated more training efforts on computational genomics, to help build genomics capacity and advance research on AA genomics. Since September 2023, our work has been further empowered by securing additional funding support from the NHGRI Diversity Center for Genome Research (DCGR), Chan Zuckerberg Biohub, in addition to the National Institute of Dental and Craniofacial Research (NIDCR) and Artificial Intelligence/Machine Learning Consortium to Advance Health Equity and Researcher Diversity (AIM-AHEAD) Southeast Hub. These funding sources enabled us to expand training scope significantly in phase two of the project.

This paper presents the progress and accomplishments of this exciting project during this period that aimed at enhancing data science and genomics capacity and workforce diversity at MMC. By outlining the strategies implemented and detailing the milestones reached, this paper highlights the significant steps taken towards addressing MMC’s urgent need to enhance research capacity and workforce diversity.

## Materials and Methods

The specific aims of the 2nd phase of this project, which were adjusted from those in year one, were to: (1) Enhance MMC’s research capacity by providing data science and computational genomics training to the community; (2) Continue to foster the collaborations between MMC’s data scientists and non-computing researchers; and (3) Assess the learning and data analytics skills of the MMC investigators, postdoctoral fellows, scientists, and/or graduate students and MMC’s research capacity enhancement.

Unlike the year one of the project, in which all our activities were conducted virtually (on Zoom or MS Teams) due to the COVID-19 pandemic [20,24], the end of the pandemic in year two enabled us to engage one another more easily to build valuable connections. All the training sessions we organized in phase two were done in-person in the classroom. However, some activities including seminars and meetings were still conducted virtually to make them manageable. All our meetings, workshops, and seminars were open to the entire MMC community and attendance was encouraged, but not mandatory.

In phase two of the project, we continued to use a web-based survey (Microsoft or Google Forms) to take advantage of its flexibility. Surveys were typically disseminated immediately after the conclusion of data science training, seminar, or workshops. On the quantitative data collected, descriptive analyses, including percentages and frequencies, were performed. The computing needs of the community and learning outcomes collected using the surveys were reviewed and assessed by the PI and Co-Investigators and presented in section below.

## Results

To support the aforementioned project aims, our activities in phase two were focused on: (1) designing short data science and genomics modules for medical and dental curriculums, (2) organizing seminars, tutorials, and workshops to promote data science training and applications, (3) continuing to foster collaborations between non-data science investigators and data science faculty, (4) developing and conducting surveys of the MMC investigators, postdoctoral fellows, staff scientists, and students, to assess their needs as well as learning outcomes. Below is a summary of our progress in achieving these project objectives.

### Data Science and Genomics Capacity Building Through Training

To enhance MMC’s research capacity, we provided data science training to the community in year one through the courses offered by MMC’s School of Applied Computational Sciences (SACS). Our assessment of trainees’ learning showed that all the trainees successfully completed the training courses [20,24]. Their overall performance as measured by course grades was excellent with none receiving a grade lower than B. This indicates that they had acquired the necessary data science skills through the training. The enrolled trainees comprised of more than 50% African Americans (AA) and 60% females.

With the great need for and success of our training, our President and CEO, Dr. James Hildreth, was committed to supporting 20 Meharry medical students in taking three SACS data science courses each per semester, starting in the Fall of 2022. As most medical students were unable to take separate data science courses due to time constraints, in year 2 we revised our strategy and goal by adopting diverse and targeted training approaches and formats. Since the SACS focuses on training graduate students for careers in academia and industry through 15-week semester long courses, we specifically focused our training efforts on designing short data science modules relevant to medical/dental practice [25], so that they fit readily into medical and dental curriculums.

In Spring 2023, we collaborated with MMC’s School of Dentistry (SOD) on the design of a computational genomics lecture for a course Introduction to Clinical Research, which is offered yearly to first-year dental students. We delivered the lecture to 72 and 74 dental students in June 2023 and 2024, respectively. In addition, we created a training module to teach dental students AI/ML and Deep Learning and delivered it in Biomedical Integration Seminars on November 8, 2023 to all (~250) dental students (including some faculty). Besides basic concepts, these two data science modules provided exercises to help students apply their learning to real-world problems. It may be worth noting that 80% of the dental students and residents in SOD are African Americans (AA).

**Figure 1:**
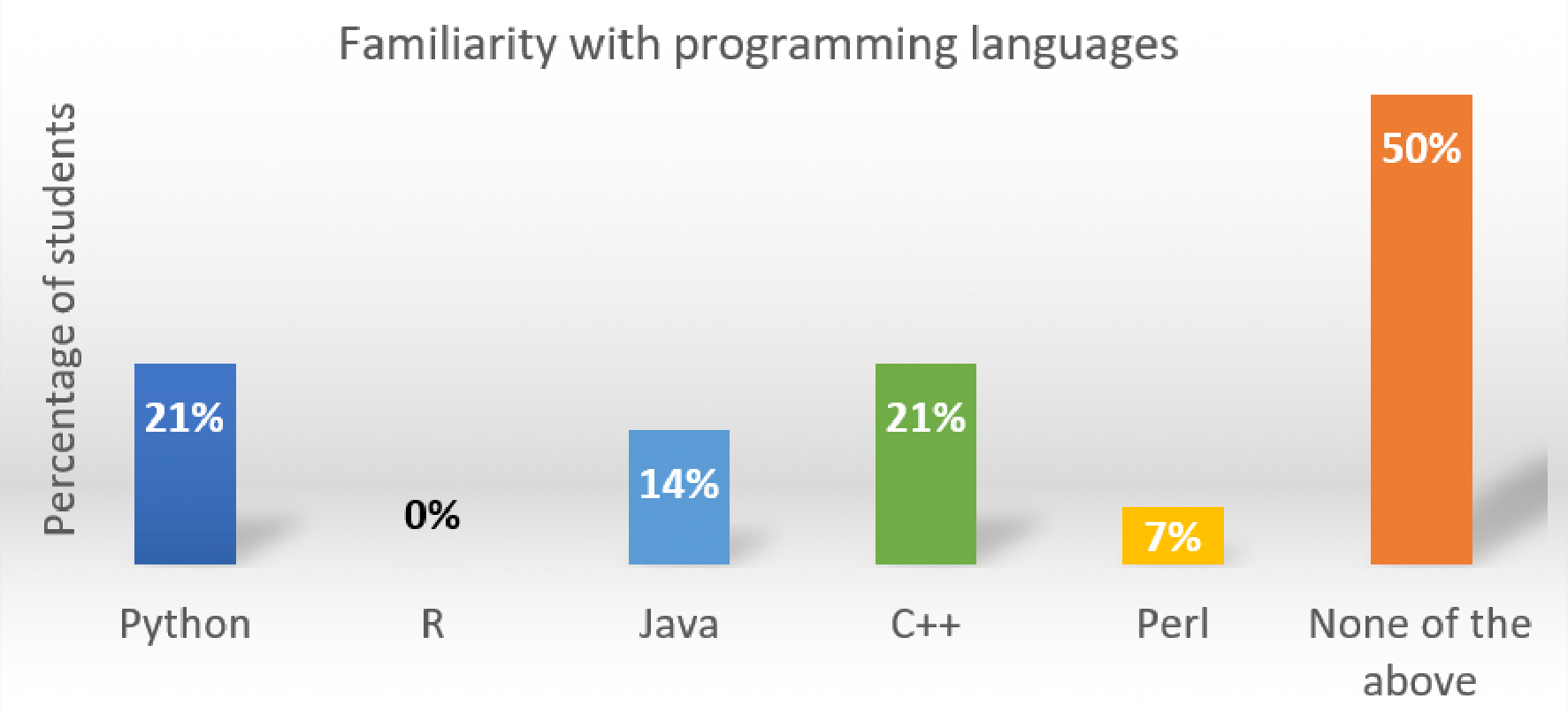
Dental students’ familiarity with programming languages.

For each training module, we conducted a survey prior to course delivery. Figure 1 shows dental students’ responses to a question in the survey regarding their computer programming skills. It shows 50% of them have no computer programming experience. Because of this, these training modules did not give any programming assignments, which are essential for students to delve into computing concepts. Besides programming skills, we also collected other information to guide lecture design.

### Seminars, Tutorials, and Workshops to Promote Data Science Trainings and Applications

In addition to short training modules, we organized seminars, tutorials, and workshops in phase 2 of the project to complement the training mentioned earlier and additionally promote applications of data science and genomics to health disparity research. Another reason was that many students and investigators expressed their interest in advanced topics about AI/ML and genomics and we hoped to address their needs through these activities.

**Figure 2:**
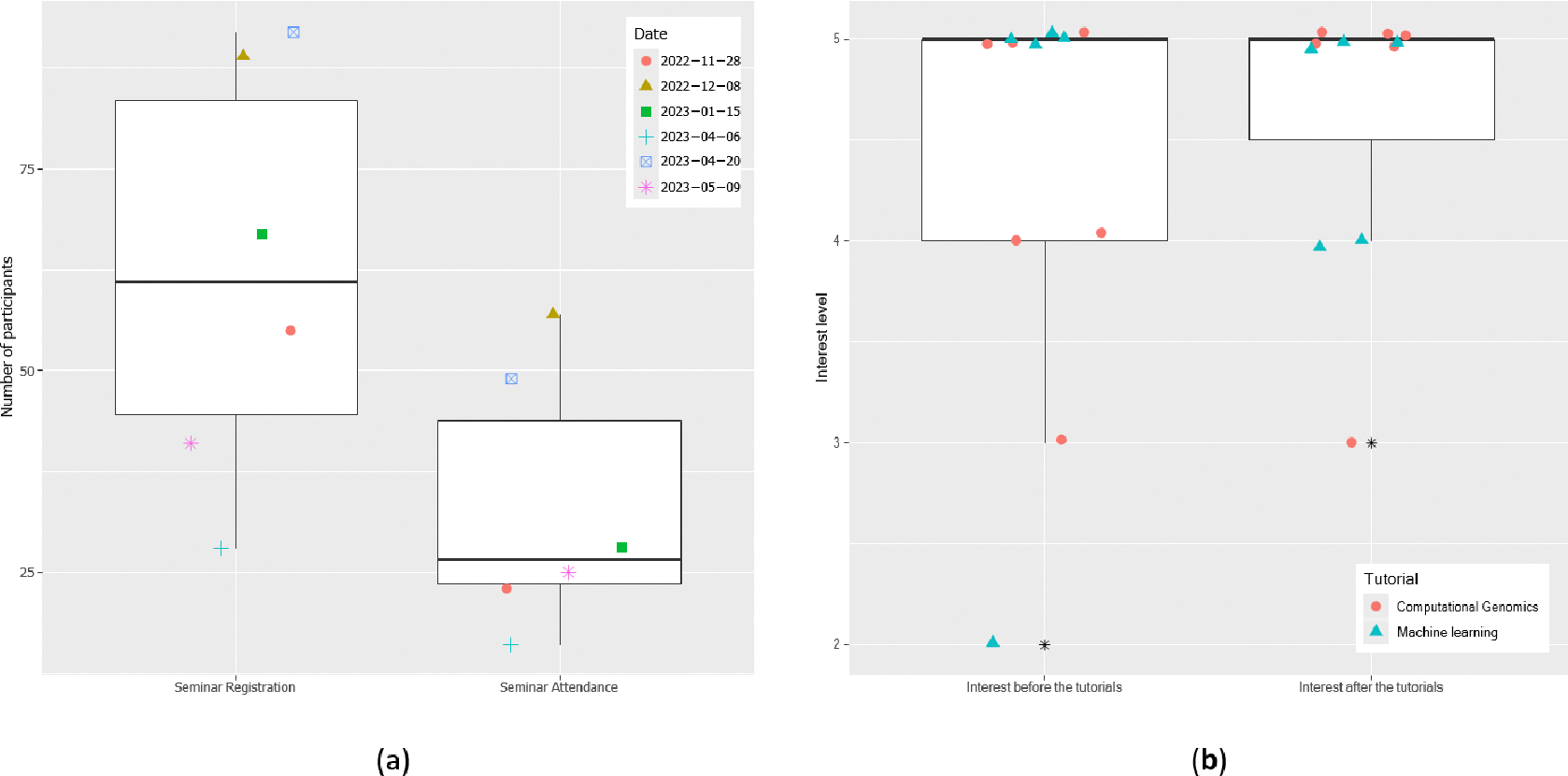
Information about our data science seminars and tutorials (see Table 1 for details). (a) Seminar registration and attendance. The y-axis of each dot in the boxplot indicates the number of registrations or attendees for a specific seminar. (b) Impact of tutorials on attendees’ interest in data science. Each point in the boxplot represents a tutorial attendee’s interest level, rated on a scale from 1 to 5. Outliers are marked with asterisks. Jittering was applied to data points in both (a) and (b) to avoid overlap.

Table 1 lists the data science seminars and tutorials we organized between November 2022 and Summer 2023. The first three seminars / tutorial in Table 1 covered the topic of computational genomics, whereas the next four were dedicated to AI/ML and their applications to clinical data analysis. The last one presented a drug discovery study at Vanderbilt University that targeted oncogene MYC through a transcription factor WDR5. Our monthly seminars and tutorials were very successful, with an average of 62 registrations and 33 attendees (Figure 2(a)). They not only exposed faculty, trainees, and students to the forefront of research but also promoted applications of data science and genomics to health disparities research.

For the two tutorials in Table 1, information and content were delivered through a mix of lecture presentations and interactive lab sessions, which followed the lecture to provide trainees with hands-on experience. A survey was conducted after the tutorials to assess trainees’ learning. The surveys showed that 70% of the attendees were faculty members and 20% were graduate students. Two questions in the surveys were about trainees’ interest in tutorial topics. A Likert scale from 1 to 5 was used to measure interest levels. The information collected from the attendees is provided in Figure 2(b), which indicates that the tutorials overall enhanced their interest in applying computational genomics and machine learning to research. A Mann-Whitney U test was performed to measure the change of interest [19], yielding a p-value of 0.60. The increase in interest level did not reach statistical significance, likely due to the small sample size as well as participants’ already high interest in data science prior to the tutorials.

The multitude of surveys conducted by us revealed a clear gap between data science and other programs on campus: MMC researchers and students need data, infrastructure, and analytical support to benefit from big data or data-intensive collaborations, whereas data scientists need knowledge about basic science, medical, and clinical practice to assist other researchers [20,24]. To narrow this gap, we organized a half-day workshop on AI/ML in August 2022. Fifty-seven researchers and students (out of 130 registrants) attended the workshop, indicating the great interest of the community in data science. Furthermore, under the additional funding support of a Biohub grant recently, we organized a two-week in-person metagenomics workshop in May 2024 to train medical / graduate students, postdoctoral fellows, staff scientists and faculty metagenomics skills. The workshop’s 1^st^ week (May 13–17^th^, 2024) taught wet lab methods and the 2^nd^ week (May 20–24^th^) taught bioinformatics and data analysis. This workshop equipped trainees with hands-on skills and research experience through lectures, labs, and journal clubs.

**Figure 3:**
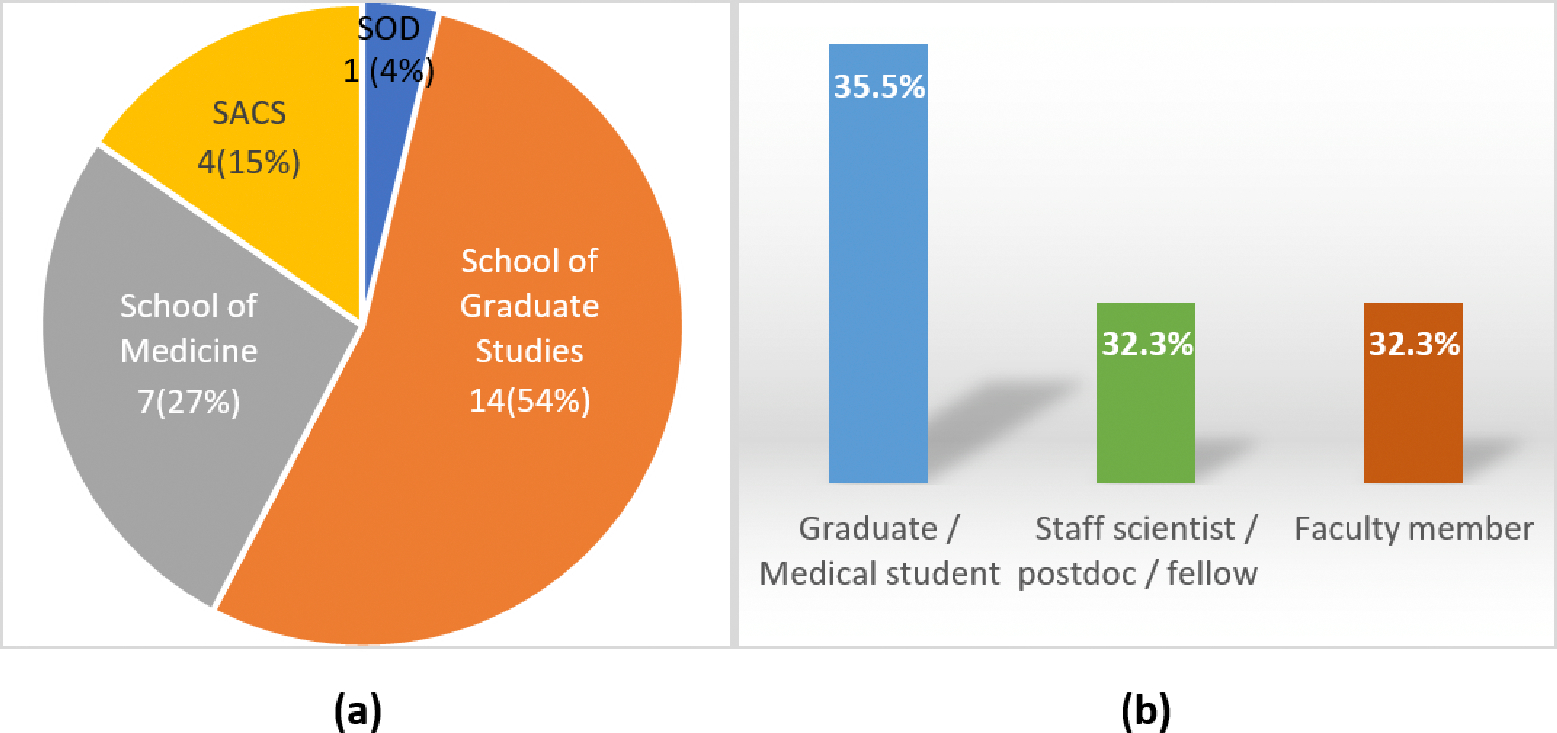
The composition of metagenomics workshop participants: (a) The affiliations of 26 Meharry attendees; (b) The roles and ratios of all 31 workshop attendees.

It may be worth mentioning that although this project was initially focused on MMC’s RCMI program, our meetings, seminars, workshops, and training were open to the entire campus, as reflected by the broad participation of the community in this project. Take the metagenomics workshop as an example. Out of the 31 workshop attendees, 26 are from MMC, as shown in Figure 3(a), among which 7 are from the School of Medicine (SOM), 14 from the School of Graduate Studies (SOGS), 1 from the SOD, and 4 from the SACS. The five non-MMC attendees are from Tennessee State University, University of Hawaii, Virginia Polytechnic Institute and State University, Morehouse School of Medicine, Connecticut Department of Public Health, respectively, highlighting a great interest in our workshop in the broad and diverse community. Furthermore, 51.6% of the attendees are females, 35.5% are AA. Additionally, among the 31 attendees, as shown in Figure 3(b), 11 (35.5%) are graduate or medical students, 10 (32.3%) are postdoctoral fellows or staff scientists, and 10 (32.3%) are faculty members (we also trained the trainers).

### Collaborations Between Data Scientists and the MMC Researchers Established

Following our practice in year one [20,24], we continued offering mini grants ($15,000 each) as subawards in phase 2 to consolidate the collaborations established in the preceding year. In addition to scientific merits, we requested each mini-grant application must meet two requirements: (a) each research proposal must be led by two principal investigators: one data science faculty and a non-data science researcher; and (b) the proposal should demonstrate the potential to promote health equity or enhance MMC’s data science capacity. In addition, applicants were highly encouraged to engage minority students in research to train next-generation data scientists.

In total seven groups applied and five were selected to receive the mini grants. Table 2 shows the titles of the five applications awarded and the last column provides the number of students engaged, which indicates the contributions of these projects toward building a diverse data science workforce through engaging minority students in research.

Our efforts to foster partnerships and promote data science and genomics in health disparities research were fruitful, and this is reflected in our successful applications for external grants. Table 3 below shows recently funded proposals submitted by us and our collaborators. The first one in Table 3 is a five-year $2.9M grant funded by the NIDCR, in which data scientists serve as Co-Investigators (Co-I) to teach dental students data analysis and computational genomics. The second one, entitled “Diversity Center for Genome Research at Meharry Medical College” received $7.0M in funding from the NHGRI. It also engages a data scientist as a Co-I to help with genomic data analysis and co-lead a Genomic Workforce Development Core. These external grants are clear evidence that: (1) the long-term partnerships between data scientists and MMC investigators have been established; and (2) this project has enhanced MMC’s data science and genomics capacity.

## Discussion

This paper presents the phase-two efforts of our project awarded initially by NIMHD, which aimed to enhance the data science capacity of Meharry Medical College (MMC) by developing a diverse data science workforce and fostering partnerships between data scientists and investigators in other disciplines. As reported in this paper, significant progress has been made in accomplishing the project aims.

The number of students engaged in our data science training in year one was small due to tight project timeline, limited resources, and our shorthandedness at the time. In phase 2, we adjusted the training strategy by focusing on designing short genomics and data science modules for medical and dental curriculums and this allowed us to significantly expand training scope. We also organized seminars, webinars, tutorials, and workshops to enhance data science and genomics applications to basic science, biomedical fields, and health disparities research and hone students’ data analyst skills. Another effort taken towards achieving project goals was fostering collaborations between data scientists and investigators from non-data science disciplines.

One limitation of our data science modules is that they focused on basic concepts without giving students any programming assignments. For students interested in advanced skills, however, programming may be needed. The use of GitHub (a web-based interface that allows developers to create, manage, and share their codes through collaboration) to share code snippets, datasets, and lecture materials could also enhance students’ learning by allowing them to download materials and learn to collaborate on projects. Another limitation of this project is that it focuses on MMC, an HBCU, which might affect the generalization of our findings to other institutions.

## Conclusions

With data analyst skills becoming increasingly crucial for leveraging big data to gain a competitive edge, accelerate scientific discoveries, and advance public health [1], data science has undergone tremendous growth in the past decade. Its widespread applications have transformed industries, the economy, health care, and scientific discovery. Accordingly, the inequitable representation of minority groups in the field, coupled with the lack of resources and infrastructure in minority-serving institutions, has become an urgent issue in the U.S.

This paper presented the phase-two efforts of our project that aimed to address the growing need to develop a diverse data science and genomics workforce and incorporate data science into health disparities research at Meharry Medical College (MMC). The computational genomics and machine learning modules that we designed were delivered to >70 and ~250 dental students, respectively, indicating that integrating data science concepts into medical curriculums was a practical approach to teaching data science to medical/dental students, whose rigorous schedules are filled with essential coursework, clinical rotations, exams, and other healthcare obligations, and, thus, prevent them from participating in training that demand a significant amount of time. Moreover, the multiple grants awarded recently to MMC, which involved both data scientists and other MMC researchers to work together, indicate that we have successfully established the long-term collaborations between data scientists and researchers in basic science and biomedical fields and that this project has enhanced MMC’s data science and genomics capacity. We hope that our experience learned as reported in this paper can be replicated by other minority- serving programs interested in enhancing their research capacity and workforce diversity at their institutions.

## Figures and Tables

**Table 1:** A list of data science seminars and tutorials organized by us in phase 2 of the project between November 2022 and Summer 2023.

Date	Title	Speaker	Topic	Format
Nov 28, 2022	Identifying Cancer Susceptibility Genes from Bioinformatics and Multi-Omics Data Analyses	Xingyi Guo PhD Vanderbilt University	Genomics	Seminar
Dec 8, 2022	A Decade of Molecular Cell Atlases	Stephen Quake, D.Phil. Stanford University	Genomics	Seminar
Jan 24, 2023	A Tutorial on Genomics: DNA-Seq and Data Analysis	Qingguo Wang, PhD Meharry Medical College	Computational Genomics	Tutorial
Feb 16, 2023	Improving Healthcare with AI: Opportunities & Challenges Across the Spectrum of Care	Jenna Wiens, PhD University of Michigan	AI/ML	Seminar
Mar 7, 2023	Machine Learning Essentials for Biomedical Data Science	Hoang Nguyen, PhD Meharry Medical College	AI/ML	Tutorial
Apr 6, 2023	Exploring Multimorbidity in EHRs: Implications for Personalized Medicine	Yaomin Xu, PhD Vanderbilt University	Statistical modeling	Seminar
Apr 20, 2023	Representing and Utilizing Clinical Textual Data for Real World Studies: An OHDSI Approach	Hua Xu, PhD Yale University	AI/ML	Seminar
May 9, 2023	Targeting MYC through WDR5	William P. Tansey, PhD Vanderbilt University	Translational research	Seminar

**Table 2:** Collaborative projects selected for mini grants by our review committee.

Project title	Junior faculty as PI	Type of application	# Students engaged
The role of circRNAs in prostate cancer malignancy and disparities	Yes	Renewal	0
A retrospective study to examine the correlation between high cholesterol and substance use disorder	No	Renewal	2
Understanding BRCT-domain containing protein function in cellular signaling using network data science on multi-omics and interaction data	Yes	Renewal	5
Role of S. cristatus in modulation of oral microbiome	No	Renewal	0
Quantitating biological cells from microscopy images using deep learning	Yes	New	1

**Table 3:** Our collaborative grant applications that were selected to receive funding recently.

Grant Agency	Title/Description	Grant Period
NIDCR	Multidisciplinary Practice-Based Research Training in Meharry’s School of Dentistry	9/13/23 – 9/12/28
NHGRI	Diversity Center for Genome Research (DCGR) at Meharry	9/21/23 – 8/31/30
Silicon Valley Community Foundation	Chan Zuckerberg Biohub Metagenomics Workshop at Meharry Medical College	Nov. 2023 – Jul. 2024
HRSA	Institution research collaborative grant on maternal health and substance use	9/30/23 – 9/29/28
NIH	AIM-AHEAD Consortium Development Projects to Advance Health Equity	9/17/23 – 9/16/25
